# Age as an Element of Diversity: Intergenerational Discussion to Improve Age-Related Attitudes

**DOI:** 10.1093/geroni/igaa057.1715

**Published:** 2020-12-16

**Authors:** Lisa Wagner, Tana Luger

**Affiliations:** 1 University of San Francisco, San Francisco, California, United States; 2 Center for the Study of Healthcare Innovation, Implementation and Policy, Greater Los Angeles VA, Los Angeles, California, United States

## Abstract

All generations must work together solving societal problems, yet age-related stereotypes are used to divide generations. Age derogation motivates younger people to vote by creating fear of an older White voting generation (Dear young people, don’t vote; 2018), and to belittle older people (“Okay, Boomer…”). Demonizing older people creates prejudice within families asking that people target loved ones, for example, by pitting educational funding for young against health funding for older adults. Neither group wins when divisiveness occurs. Generation to Generation, an intergenerational course for older and younger adults, promotes intergenerational contact. Students discuss topical issues (e.g., racism) in multi-generational groups. Using pretest-posttest design, all students were invited to complete questionnaires at beginning and end of term. Younger adults reported significant increases in affection, comfort, kinship, engagement and enthusiasm for older adults, whereas older adults showed stability over time. Intergenerational discussion may facilitate improved connections between generations.

